# Digging Its Own Site: Linear Coordination Stabilizes a Pt_1_/Fe_2_O_3_ Single-Atom Catalyst

**DOI:** 10.1021/acsnano.4c08781

**Published:** 2024-09-18

**Authors:** Ali Rafsanjani-Abbasi, Florian Buchner, Faith J. Lewis, Lena Puntscher, Florian Kraushofer, Panukorn Sombut, Moritz Eder, Jiří Pavelec, Erik Rheinfrank, Giada Franceschi, Viktor Birschitzky, Michele Riva, Cesare Franchini, Michael Schmid, Ulrike Diebold, Matthias Meier, Georg K. H. Madsen, Gareth S. Parkinson

**Affiliations:** † Institute of Applied Physics, TU Wien, Vienna AT 1040, Austria; ‡ Institute of Materials Chemistry, TU Wien, Vienna AT 1060, Austria; § Faculty of Physics and Center for Computational Materials Science, University of Vienna, Vienna AT 1090, Austria; ∥ Dipartimento di Fisica e Astronomia, Università di Bologna, Bologna IT 40126, Italy

**Keywords:** adsorption site, platinum, hematite, iron oxide, single-atom catalysis

## Abstract

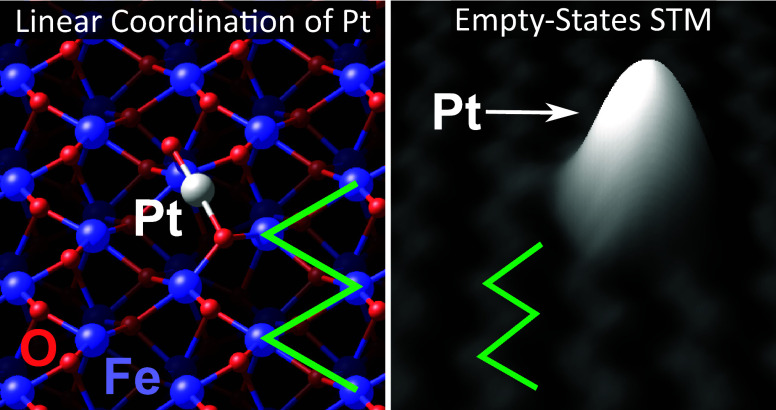

Determining the local coordination of the active site is a prerequisite for the reliable modeling of single-atom catalysts (SACs). Obtaining such information is difficult on powder-based systems and much emphasis is placed on density functional theory computations based on idealized low-index surfaces of the support. In this work, we investigate how Pt atoms bind to the (11̅02) facet of α-Fe_2_O_3_; a common support material in SACs. Using a combination of scanning tunneling microscopy, X-ray photoelectron spectroscopy, and an extensive computational evolutionary search, we find that Pt atoms significantly reconfigure the support lattice to facilitate a pseudolinear coordination to surface oxygen atoms. Despite breaking three surface Fe–O bonds, this geometry is favored by 0.84 eV over the best configuration involving an unperturbed support. We suggest that the linear O–Pt–O configuration is common in reactive Pt-based SAC systems because it balances thermal stability with the ability to adsorb reactants from the gas phase. Moreover, we conclude that extensive structural searches are necessary to determine realistic active site geometries in single-atom catalysis.

## Introduction

Single-atom catalysis (SAC) has emerged as one of the hottest topics in catalysis research in recent years.^1−4^ While the motivation was initially to minimize the amount of precious metal required for heterogeneous catalysis,^1^ it quickly became clear that “single atoms” have properties distinct from their parent metal.^5^ Such species typically bind to the surface oxygen atoms on metal oxide supports and become cationic.^6^ The coordination of the metal adatom and the geometry of the bonds affect its oxidation state and, with it, the binding strength of reactants and the catalytic activity.^7−9^ This suggests that the reactivity could be tuned if the active site geometry could be controlled. Unfortunately, it remains difficult to determine the atomic-scale details of the active species on a powder support, let alone control it. As a consequence, catalytic reactions are typically modeled computationally, assuming that a metal atom occupies a favorable site on a low-index facet of the support material or a substitutional cation site within it.

An alternative approach to study fundamentals in SAC is to utilize model single-crystal supports prepared under ultrahigh-vacuum conditions, where the structure can be precisely determined.^10−13^ This approach represents a natural complement to theory and can be used to ascertain the types of active sites that occur and to explore them at the atomic level.^13^ In this paper, we select a classic system in SAC, Pt_1_ supported by iron oxide, as utilized by Qiao et al. in their pioneering 2011 study.^1^ That study^1^ and many subsequent works^14−17^ have utilized a bulk-truncated α-Fe_2_O_3_(0001) surface as the basis for the computational modeling of the system. However, that surface is complex, and its structure remains controversial.^18−24^ We instead opt for the (11̅02) facet, which is similarly prevalent in nanomaterials.^25^ Crucially, the surface presents a simple, bulk-truncated structure after UHV preparation,^26−29^ and the small unit cell provides a solid basis for computational modeling. The choice of this facet is further motivated by the recent works by Gao et al.,^30,31^ who synthesized α-Fe_2_O_3_(11̅02) nanocubes and found Pt and Pd atoms to be both stable and highly active for the electrochemical oxygen reduction reaction (ORR) and alkyne semihydration, respectively. Here also, a Pt atom bound to a bulk-truncated surface was used as the basis for the computational modeling.^30,31^ Using a combination of surface science techniques and an extensive computational search, we show that Pt atoms are stable at room temperature on α-Fe_2_O_3_(11̅02) because they create a site in which Pt is 2-fold coordinated to lattice oxygen atoms. This configuration is 0.84 eV more stable than any configuration that can be achieved assuming an intact bulk-truncated surface,^30,31^ even though it breaks three Fe–O bonds in the support. We conclude that the unusual linear Pt configuration is likely advantageous for Pt single-atom catalysts because its undercoordination makes it reactive to atoms in the gas phase. The configuration involves significant modification of the oxide support, which is hard to envision based on chemical intuition alone. As such, our work exemplifies that extensive structural searches are required to determine the optimal active site configurations.

## Results and Discussion

**Figure 1:**
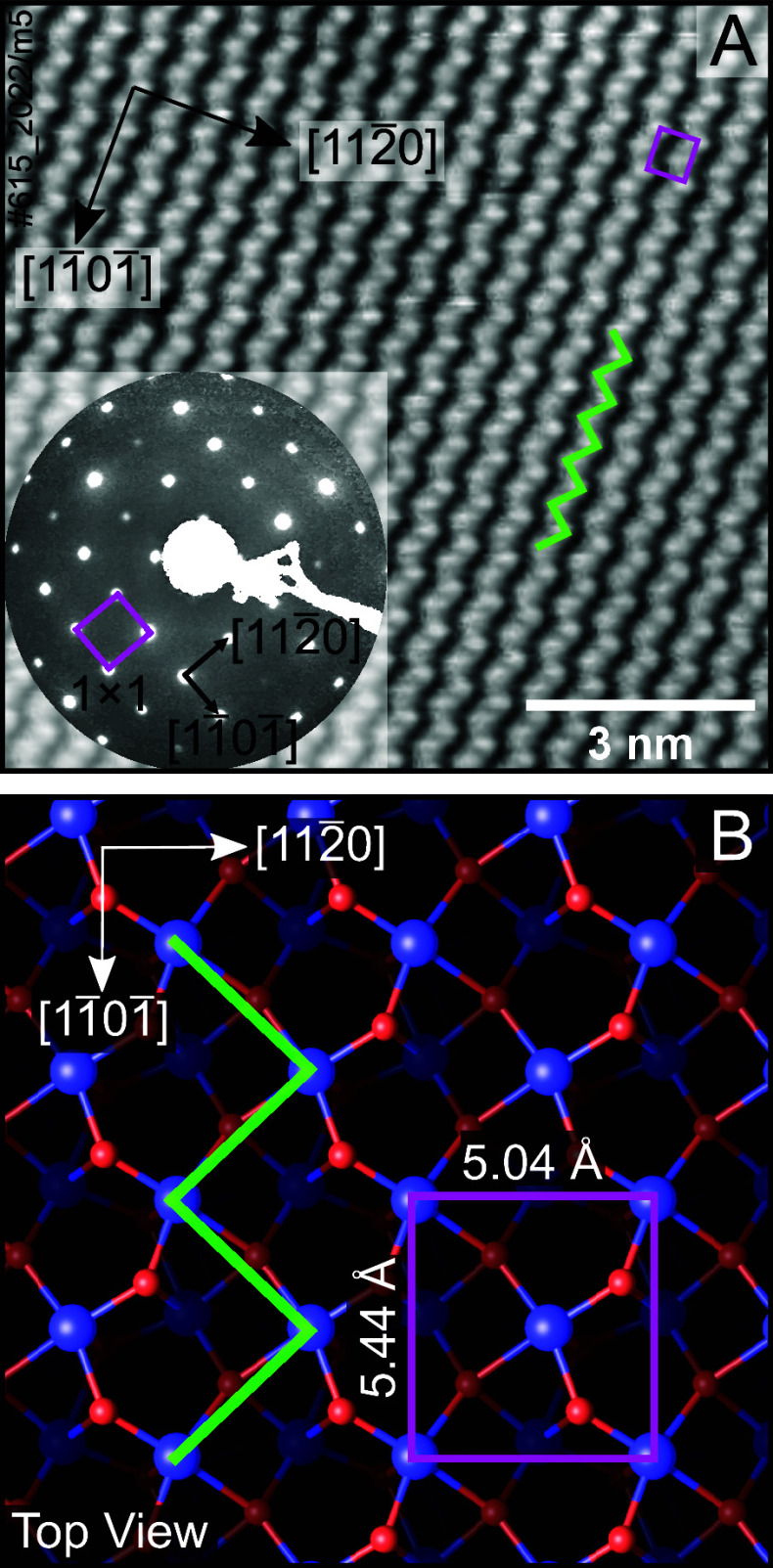
Pristine Fe_2_O_3_(11̅02)-(1 × 1) surface. (A) STM image acquired at room temperature (*U*_sample_ = +3 V, *I*_tunnel_ = 0.3 nA). Surface Fe atoms appear bright in empty states. Inset: Corresponding LEED pattern (150 eV), which shows the (1 × 1) periodicity. (B) Top view of the Fe_2_O_3_(11̅02)-(1 × 1) surface with the unit cell marked in pink (5.04 × 5.44 Å^2^). Fe^3+^ cations are shown as blue spheres, while oxygen atoms are red. The zigzag rows of iron running in the [11̅01̅] direction are highlighted in green.

Figure 1 displays an STM image obtained from the UHV-prepared, pristine Fe_2_O_3_(11̅02)-(1 × 1) surface after UHV preparation.^26,29^ The STM image was acquired with a positive sample bias and reveals the local density of empty states. Since the bottom of the conduction band is dominated by Fe 3d states,^26^ the image displays bright zigzag rows of iron atoms running in the [11̅01̅] direction. The LEED pattern shown in Figure 1A (inset, acquired with a 150 eV electron energy) shows a (1 × 1) periodicity, consistent with a bulk-truncated surface.^26,29,32^Figure 1B shows a top view of the Fe_2_O_3_(11̅02)-(1 × 1) surface structure determined by DFT. This surface has a near-square unit cell of 5.04 × 5.44 Å^2^, indicated by the pink rectangle. Note that surface oxygen atoms have a 3-fold coordination, compared to four in the bulk, and surface iron atoms have a 5-fold coordination, compared to six in the bulk.^26^ A larger-area STM image is presented in Figure S1.

**Figure 2:**
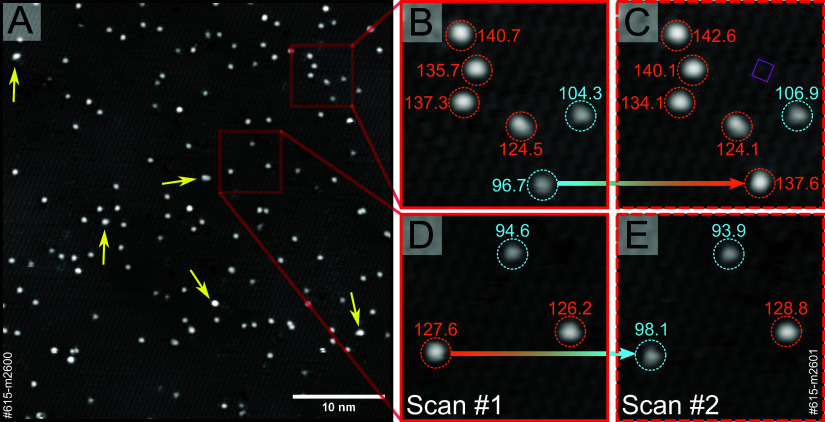
(A) STM image (*U*_sample_ = +3.0 V, *I*_tunnel_ = 0.35 nA) of 0.005 ML Pt vapor-deposited on Fe_2_O_3_(11̅02)-(1 × 1) at room temperature. The yellow arrows indicate some larger Pt agglomerates. (B–E) Sequential STM images showing enlarged squares drawn in (A). The pink rectangle drawn in (C) represents an Fe_2_O_3_(11̅02)-(1 × 1) unit cell. The numbers next to each of the deposited single atoms indicate the apparent height of that atom in picometers relative to the substrate. The two different sets of apparent heights are marked by cyan and orange circles.

Figure 2A presents an STM image acquired at room temperature following the deposition of a nominal coverage of 0.005 monolayer (ML) Pt onto the pristine Fe_2_O_3_(11̅02)-(1 × 1) surface. Compared to the clean surface, near-circular protrusions are seen with a density of ∼0.006 ML, consistent with isolated Pt_1_ atoms. A few larger protrusions are likely to be aggregates of more than one atom. These are highlighted with yellow arrows in Figure 2A. Interestingly, the apparent height of the features assigned as individual atoms is not uniform. While the majority of them have an apparent height of 130 ± 10 pm (orange circles in Figure 2B–E), ∼20% have an apparent height of 100 ± 10 pm (cyan circles). Figure 2B–E shows that the apparent height can fluctuate between sequential images of an STM movie. In Figure 2B,C, a protrusion changes from low to high, and in Figure 2D,E from high to low. This behavior is typical for the adsorption and desorption of adsorbates. Such events are rare in the 5 × 10^–11^ mbar residual gas, as seen by STM measurements on the same spot on the sample taken over the course of 2 h, displayed as a time-lapse STM “movie” (the full movie is included in the Supporting Information as Movie S1). Based on the extremely low residual gas pressure and the prevalence of high to low transitions, we conclude that the more numerous higher protrusions are bare Pt atoms, while the lower species have adsorbed a molecule from the residual gas. Movie S1 also shows that the system is stable with no diffusion of the Pt-related species. Some mobile species are observed, but we attribute these to adsorbed water/OH groups (we have previously shown that water partially dissociates on Fe_2_O_3_(11̅02)-(1 × 1) and desorbs at approximately 340 K^32,34^).

**Figure 3:**
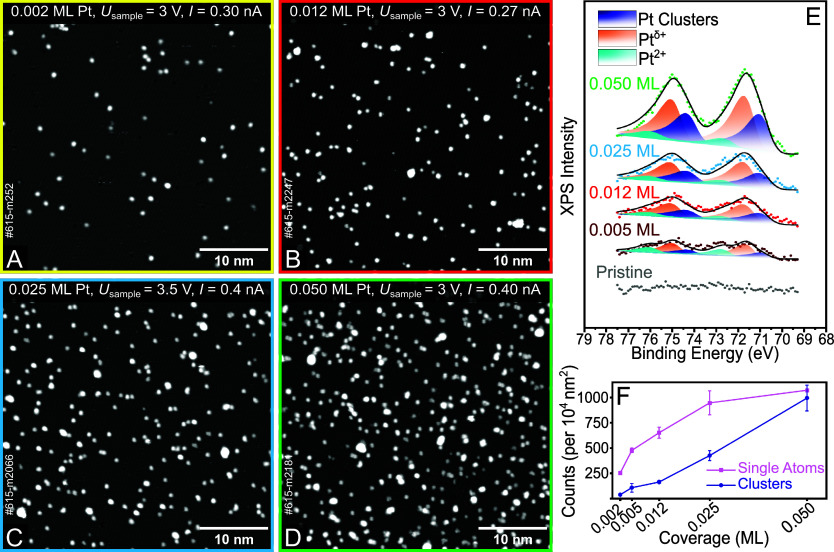
Characterizing different surface coverages of Pt on pristine α-Fe_2_O_3_(11̅02)-(1 × 1) at room temperature. (A–D) STM images taken at room temperature for Pt coverages of 0.002, 0.012, 0.025, and 0.050 ML. (E) XPS spectra of the Pt 4f region (Al Kα, a 70° grazing emission, with a pass energy of 16 eV) for UHV-prepared pristine Fe_2_O_3_(11̅02)-(1 × 1), as well as 0.005, 0.012, 0.025, and 0.050 ML as-deposited Pt. (F) Plot showing the density of single atoms and clusters at various Pt surface coverages. The error bars shown are one standard deviation (1σ).

In Figure 3, we present STM images obtained after vapor-depositing Pt at four different coverages (0.002, 0.012, 0.025, and 0.050 ML) onto the pristine UHV-prepared Fe_2_O_3_(11̅02)-(1 × 1) surface at room temperature. The circular features assigned as single atoms remain present at all coverages, in addition to increasingly numerous and increasingly large agglomerates. Since the bare Pt atoms do not diffuse at room temperature, cluster formation is likely a consequence of incoming Pt atoms arriving in close proximity to Pt on the surface. In Figure 3F, we show the density of adatoms and clusters extracted from several different images at the different coverages. The number of adatoms increases rapidly initially but saturates due to the formation of coexisting Pt clusters. At the highest coverage studied here, the surface hosts a similar density of adatoms and clusters. STM images conducted after postannealing (not shown) suggest that the onset of thermal sintering occurs at ∼150 °C.

We also acquired XPS data from the surfaces shown in Figures 2 and 3B–D. The spectra were obtained at room temperature immediately after Pt was deposited onto the surface. The lowest coverage where a meaningful Pt 4f XPS spectrum was possible in our setup was 0.005 ML. In this case, the Pt 4f signal is broad, indicative of the presence of more than one component. The data can be consistently fitted using three sets of doublets, which is reasonable given the clear observation of adatoms with and without adsorbates and Pt clusters. The fit utilized an asymmetric Lorentzian/Gaussian function, and all peaks were constrained to have the same full width at half-maximum (optimized value of 1.1 eV). The Pt 4f_7/2_ component for Pt clusters appears at 71.2 eV (dark blue in Figure 3E). This reference value was confirmed by fitting the Pt 4f XPS spectrum of a thermally sintered Pt/Fe_2_O_3_(11̅02)-(1 × 1) sample (at 500 °C, see Figure S2) with a single doublet. A second component appears with a binding energy of 71.8 ± 0.1 eV (orange). The growth of this peak with coverage suggests that it is due to the “high” Pt adatoms observed in Figure 3A–D. Since it appears at a higher binding energy than the Pt clusters, we assign these species as Pt^δ+^. The small peak at 72.7 ± 0.1 eV (cyan) is in the energy range usually reported for Pt^2+^.^30,35−37^ To confirm that adsorption from the residual gas is responsible for this peak, we intentionally exposed the as-prepared model catalysts to both H_2_O and O_2_ in separate experiments (see Figure S3). Both experiments led to an increase of the Pt 4f_7/2_ peak to 72.7 eV, as expected. Without these additional adsorbates, the peak at 72.7 ± 0.1 eV does not increase significantly with Pt coverage. It is constant because the background pressure is low and the time between Pt deposition and the XPS measurement is similar in all experiments.

**Figure 4:**
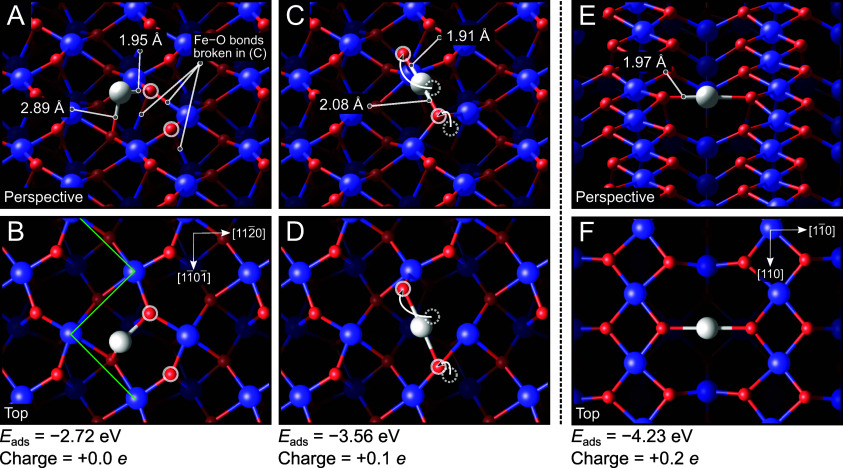
Perspective and top view of two of the lowest-energy configurations found for Pt_1_ on α-Fe_2_O_3_(11̅02)-(1 × 1) during the structural search, following optimization by DFT. (A,B) Pt adsorbed on an unreconstructed surface. The Pt atom has one strong bond to a surface O atom and one weak bond to a subsurface O atom. The three Fe–O bonds that will be broken by the reconstruction are indicated. (C,D) Surface reconstruction induced by Pt. One O atom moves atop a surface Fe atom to facilitate a pseudolinear bonding configuration for the Pt atom. Adsorption energies (*E*_ads_) are given with respect to a single Pt atom in the gas phase, and Pt Bader charges are indicated. Dashed white circles indicate the prior positions of the oxygen atoms to which the Pt binds. (E,F) Minimum energy configuration of the Pt_1_/Fe_3_O_4_(001) system, as determined previously,^13^ which also exhibits a 2-fold coordination to oxygen. Oxygen atoms are red, iron blue, and platinum white.

To determine the lowest-energy configurations of Pt atoms on the α-Fe_2_O_3_(11̅02)-(1 × 1) surface, we performed an extensive computational structure search, employing the covariance matrix adaptation evolution strategy (CMA-ES) algorithm^16,38^ using the Clinamen2 package.^39^ Details of the methodology are provided in the computational details. The optimal geometry for a Pt atom on an unperturbed surface is shown in Figure 4A,B. The Pt atom binds to two O atoms, to one surface (i.e., 3-fold coordinated) O atom with a bond length of 1.95 Å, and to one subsurface 4-fold coordinated O atom with a bond length of 2.89 Å. The latter is on the edge of what might be considered a bond, so one can expect that this structure is not ideal. For reference, the adsorption energy of this configuration is −2.72 eV with respect to a gas-phase Pt atom. Binding the Pt to two surface oxygen atoms along the [11̅01̅] direction (highlighted with white circles in Figure 3A,B) would likely be more stable but is precluded by the presence of the surface Fe atoms in-between and the nonlinear O–Pt–O bond angle that would result. The optimal configuration obtained by the CMA-ES search circumvents this issue by restructuring the surface, displacing one of the surface oxygen atoms to a new site atop a surface Fe atom (as shown by the white arrows in Figure 4C,D). This breaks two bonds of this O atom, leaving it coordinated to only a single surface Fe atom and the Pt atom (Pt–O bond length of 1.91 Å). Crucially, the rearrangement facilitates a quasi-linear 2-fold geometry for the Pt atom (Figure 4C,D). The second bonding partner is lifted out of the plane, breaking a further Fe–O bond to a subsurface oxygen. The Pt–O bond length to this atom is 2.08 Å. Despite the significant rearrangement and the complete breaking of multiple Fe–O bonds, the Pt has an adsorption energy of −3.56 eV, which is an overall energy gain of −0.84 eV compared to the structure shown in Figure 4A,B. The barrier to move from the structure shown in Figure 4A–D is calculated to be 0.3–0.5 eV based on our climbing image-nudged elastic band (CI-NEB)^40^ calculations, meaning that it can easily be overcome at room temperature. STM simulations based on the structure shown in Figure 4C,D are included as Figure S4 and perfectly fit with the observed location between the zigzag rows of the underlying support.

On reflection, the structure obtained by the structural search is consistent with our previous experience studying Pt adsorption on Fe_3_O_4_(001), another model iron oxide support (see Figure 4E,F).^13^ There, a pseudolinear O–Pt–O structure was observed but attributed to the fact that this geometry is effectively a bulk continuation site on that surface, i.e., the Pt is close to the position where the next Fe would have been located if the Fe_3_O_4_ lattice were continued outward. However, the O atoms move up to allow a more linear O–Pt–O bond than could otherwise be achieved with the bulk-like spacing. On Fe_2_O_3_, the ground state obtained is strikingly similar, featuring the same 2-fold linear coordination to surface oxygen atoms with virtually the same O–Pt–O bond angle (174° vs 178°). The disparity in adsorption strength (on Fe_3_O_4_(001), Pt has an adsorption energy of −4.23 eV, stronger by −0.67 eV) can be attributed to the rearrangement of the α-Fe_2_O_3_(11̅02)-(1 × 1) surface required to create the favored adsorption site, whereas the preferred site for Pt already exists on Fe_3_O_4_(001) without incurring additional energetic cost. The electronic DOS for the bare Pt adatom and linear coordinated Pt is shown in Figure S5. The lower energy of the occupied states for the latter species is consistent with its stronger adsorption energy.

The similarity between the Pt atoms on the two surfaces also extends to their electronic properties. The d-band filling, an important parameter concerning catalytic properties,^13^ is similar, as confirmed by a Bader charge analysis. Specifically, we find Bader charges of +0.1*e* for Pt_1_/Fe_2_O_3_(11̅02)-(1 × 1) and +0.2*e* for Pt_1_/Fe_3_O_4_(001). This is in agreement with the XPS results for Pt/Fe_2_O_3_(11̅02)-(1 × 1), which show the experimentally observed species without any adsorbates at 71.8 eV, close to Pt^0^ in Pt metal.

The linearly coordinated Pt^δ+^ observed here is unusual for Pt, which is coordinated square-planar in the Pt^2+^ oxidation state (including in the bulk oxide PtO) or octahedral (6-fold) in the Pt^4+^ oxidation state (as in bulk PtO_2_). There are some examples of linearly coordinated Pt complexes in homogeneous catalysts,^101−104^ but square-planar Pt^2+^ complexes dominate. Two-fold linear coordination is favored for metal oxides with a nominal d^10^ configuration, such as Ag_2_O and Cu_2_O. In principle, one could consider a d^10^ electronic configuration for Pt^0^, but our DFT calculations indicate a 5d^9^6s^1^ occupation. It must be noted, however, that Ag_2_O and Cu_2_O also do not have a simple d^10^ occupation; this is related to strong s–d hybridization.^41−43^ Linearly coordinated Pt^0^ adatoms were previously predicted to occur on reduced CeO_2_(100),^105^ but this configuration was determined to be metastable against square-planar Pt^2+^.

The linear Pt coordination found here is important for the site’s ability to coordinate ligands: Pt^2+^ species on CeO_2_(111) are chemically inert because they are 4-fold coordinated to O^2–^ anions at step edges.^12,44−46^ Such a geometry constitutes coordinative saturation akin to a bulk Pt atom in PtO. Compared with the 4-fold Pt^2+^ configuration, the linearly coordinated Pt found here can be seen as having two free ligands, which makes it free to adsorb oxidizing species from the gas phase, as was observed during our STM experiments.

The stability of isolated Pt atoms on Fe_2_O_3_(11̅02)-(1 × 1) is also surprising because Rh (a more oxophilic metal) sinters immediately upon deposition at room temperature.^47^ This is probably because the energy gain from moving Rh to a linear configuration is insufficient to motivate the restructuring of the surface, as seen with Pt. Creating a Rh square-planar geometry on Fe_2_O_3_(11̅02)-(1 × 1) would require an even more dramatic reconfiguration of the support, or additional ligands to be provided by adsorbates. Indeed, we observed that Rh deposition in a water vapor background led to stable adatom species, which we inferred to be square-planar with two ligands provided by surface and two by surface OH groups.^48^ This additional stabilization by OH has been proposed previously for several Pt SAC systems, including Pt/ceria^49^ and Pt/anatase TiO_2_,^9^ and it is conceivable that Pt could transition between the linear and square-planar configurations during a reaction cycle. The ability to switch between different configurations underlies the performance of many homogeneous catalysts, and it would be extremely interesting if such behavior was replicated on a solid surface. We note, however, that the Pt^δ+^-Pt^2+^ redox cycle could also result in a more severe restructuring of the system, such as sintering. Finally, we note that there is no evidence for the incorporation of Pt into substitutional sites within the Fe_2_O_3_(11̅02)-(1 × 1) lattice, which was observed previously for Rh.^47^ This is likely due to the lower oxophilicity of Pt.

## Conclusions

In summary, Pt atoms rearrange the Fe_2_O_3_(11̅02)-(1 × 1) support lattice to create a pseudolinear configuration. The adatoms are stable at room temperature and readily adsorb molecules from the residual gas. The unusual coordination is ideal for SAC because it is stable enough to prevent thermal sintering, but energy can, in principle, be gained by adsorbing reactants, especially if the Pt is able to form a square-planar environment. We conclude that a linear binding coordination is likely a common motif for active Pt atoms in single-atom catalysis but that thorough computational searches for the ground state geometry are an important first step in the computational modeling of single-atom catalysts.

## Experimental and Computational Methods

All room temperature surface analysis experiments were performed in a UHV system consisting of a preparation chamber (background pressure below 10^–10^ mbar) and an analysis chamber (<5 × 10^–11^ mbar). Scanning tunneling microscopy measurements were carried out using an Omicron μ-STM operated in constant-current mode with an electrochemically etched tungsten tip. The analysis chamber is further equipped with a commercial low-energy electron diffraction (LEED) instrument, a nonmonochromatic X-ray source (Al Kα anode), and a SPECS Phoibos 100 analyzer for X-ray photoelectron spectroscopy operated at grazing emission, 70° from the surface normal.

A homoepitaxial 0.03 at. % Ti-doped hematite film grown on a natural Fe_2_O_3_(11̅02) single-crystal sample (SurfaceNet GmbH) with a miscut of <0.3° and dimensions of 10 × 10 × 0.5 mm^3^ was used as the substrate (for detailed preparation of the film, see refs (47, 50)). The sample was cleaned with multiple cycles of Ar^+^ sputtering (≈2 μA/cm^2^, 1 keV, 10 min) and subsequent annealing in oxygen at 520 °C (*p*_O_2__ = 2 × 10^–6^ mbar, 30 min). LEED patterns were acquired from the UHV-prepared surface to ensure a fully oxidized stoichiometric Fe_2_O_3_(11̅02) surface possessing a (1 × 1) bulk-terminated structure.^26^ After the substrate had cooled to room temperature, Pt deposition was performed using an Omicron single-pocket electron-beam evaporator (liquid N_2_ cooled) in the preparation chamber. The evaporation rate was calibrated by a temperature-stabilized (water-cooled) quartz crystal microbalance (QCM). The fraction of the substrate covered by Pt atoms is given in monolayers (ML), which is defined throughout this article as two Pt atoms per Fe_2_O_3_(11̅02)-(1 × 1) unit cell, or 7.3 × 10^14^ atoms/cm^2^, the same as the density of the Fe atoms in the surface.

The DFT calculations were carried out using the Vienna ab initio simulation package.^51,52^ We employed the projector-augmented wave method,^53,54^ with the plane-wave basis set cutoff energy set to 550 eV. Calculations were spin-polarized and performed with Γ-centered *k*-meshes of (4 × 4 × 1) for the 2 × 2 supercells of hematite and Γ-centered *k*-meshes of (2 × 2 × 1) for the (2√2 × 2√2)R45° supercells of magnetite. Supercells were employed to approximate low experimental Pt coverages. *k*-meshes were doubled for electronic DOS calculations.

Other details, such as the slab structures of the hematite and magnetite surfaces and input parameters, were unchanged with respect to our previous studies.^34,55^ The convergence criterion was an energy change of 10^–6^ eV for the electronic self-consistency step and forces smaller than 0.02 eV/Å. The Perdew–Burke–Ernzerhof (PBE)^56^ functional was used. An effective on-site Coulomb repulsion term^57^ was applied for the 3d electrons of the Fe atoms; *U*_eff_ = 5 and 3.61 eV for hematite and magnetite, respectively. Adsorption energies are given with respect to a single gas-phase Pt atom via the formula *E*_ads_ = *E*_surface+Pt_ – *E*_surface_ – *E*_Pt_, where *E*_ads_ is the adsorption energy of Pt, *E*_surface+Pt_ is the total energy with the Pt atom present on the surface, *E*_surface_ is the energy of the pristine surface, and *E*_Pt_ is the energy of a single Pt atom in the gas phase. CI-NEB calculations were performed at the Γ-point only, with four images between the Pt adatom and the pseudolinear-coordinated Pt configurations. Convergence was assumed when forces were below 0.02 eV/Å. The images were initialized by linear interpolation.

Pt/Fe_3_O_4_(001) has been previously investigated by a combination of experimental and DFT techniques^13^ and serves here as a comparison; thus, we have recalculated it with the same parameters as the hematite system.

For structure prediction, we used the covariance matrix adaptation evolution strategy (CMA-ES)^16,38^ to identify the minima of the potential energy surface (PES) calculated at the DFT level. The CMA-ES is a powerful general-purpose evolutionary algorithm whose recent atomic-structure-targeted implementation by the Clinamen package has been successful in exploring the PES of bulk defects,^58^ as well as surface reconstructions such as for SrTiO_3_(110).^59^ A subset of the evolutionary search runs in the present work used a newer version of our CMA-ES code, which has since been released as Clinamen2.^39^

The CMA-ES provides a way of searching the PES that is more systematic and automated and less dependent on human intuition than constructing trial structures manually. Explicit structure input is only required once at the beginning of a CMA-ES run, for defining an initial guess called the founder. The founder is a purely hypothetical structure, meant to facilitate exploration of structures without biasing the search to any preconceived “reasonable” structure. Nevertheless, our choice of founder was motivated by two concerns. First, it should facilitate the exploration of structures that see more significant embedding of the Pt into the surface than bare-adatom structures like that of Figure 4A. Second, we wanted to achieve such a founder with the minimum possible changes to a bulk-truncated surface and without introducing any further assumptions (such as from the experimental STM images or structures known from previous studies of similar systems). This founder is depicted and described in more detail in Figure S6. It was used for all CMA-ES search runs in this work. Due to the stochastic nature of the CMA-ES algorithm, different runs still explore different regions of the PES.

Running a CMA-ES search requires evaluation of the energy of a large number of trial structures. To accelerate these evaluations, which we performed with pure DFT rather than any surrogate model, they were carried out in a smaller c(2×2) supercell of Fe_2_O_3_(11̅02)-(1 × 1) and using less strict convergence criteria. The choice of *U*_eff_ (typically ±1 eV) or choice of functional (i.e., PBE or PBEsol tested here) did not affect the conclusions of our work. Promising structures encountered during CMA-ES searches were subsequently refined by DFT relaxation at the level described above. More information about the candidate selection process as well as CMA-ES search termination criteria and settings choices can be found in the SI.

## Supporting Information Available

Time-lapse movie following the deposition of Pt on Fe_2_O_3_(11̅02)-(1 × 1) with a duration of 120 min (MP4)
Large-area STM image of the pristine Fe_2_O_3_(11̅02)-(1 × 1) surface, Pt 4f XPS data acquired following annealing of Pt/Fe_2_O_3_(1102)-(1 × 1) to 500 °C and after exposure to H_2_O and O_2_, STM simulation of the restructured Pt atom (surface O displaced) on Fe_2_O_3_(11̅02)-(1 × 1), electronic density of states (DOS) of the Pt adatom and the pseudolinear-coordinated Pt, and structure model of the founder and further details regarding the CMA-ES search (PDF)
