# Supplementary material for: Digging Its Own Site: Linear Coordination Stabilizes a Pt1/Fe2O3 Single-Atom Catalyst
Source: ACS Nano. 2024 Sep 18;18(39):26920–7. doi: 10.1021/acsnano.4c08781 (PMC11447906; doi:10.1021/acsnano.4c08781)
Supplement: Supplementary file 2 — nn4c08781_si_002.pdf [file nn4c08781_si_002.pdf]

Supplementary Information:

Digging Its Own Site: Linear Coordination Stabilizes a Pt_1/Fe_2O_3 Single-Atom Catalyst

Ali Rafsanjani-Abbasi[1], Florian Buchner[2], Faith J. Lewis[1], Lena Puntscher[1], Florian Kraushofer[1], Panukorn Sombut[1], Moritz Eder[1], Jiří Pavelec[1], Erik Rheinfrank[1], Giada Franceschi[1], Viktor Birschitzky[3], Michele Riva[1], Cesare Franchini[3,4], Michael Schmid[1], Ulrike Diebold[1], Matthias Meier[1,3], Georg K. H. Madsen[2], Gareth S. Parkinson[1]*

[1]Institute of Applied Physics, TU Wien, Vienna, AT 1040, Austria

[2]Institute of Materials Chemistry, TU Wien, Vienna, AT 1060, Austria

[3]Faculty of Physics and Center for Computational Materials Science, University of Vienna, Vienna, AT 1090, Austria

[4]Dipartimento di Fisica e Astronomia, Università di Bologna, Bologna, IT 40126, Italy

[*]Corresponding Author

Gareth.parkinson@tuwien.ac.at

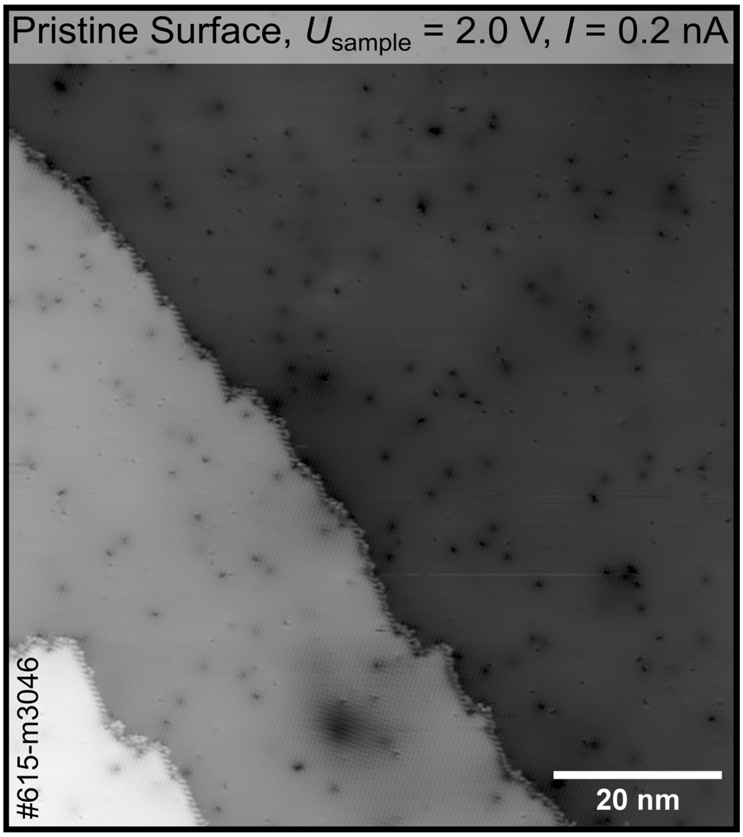

Figure S1. A large-area STM image of the pristine $Fe_2O_3(1\bar{1}02)$-(1×1) surface.

In Figure S1, terraces of the pristine hematite surface, large enough for scanning tunneling microscopy (STM), are clearly visible. Apart from a limited number of point defects, such as vacancies or the possible presence of OH ions, no other significant features are observed on the surface of these terraces. The image was corrected for distortions[1] and the contrast on the terraces was enhanced by image processing.

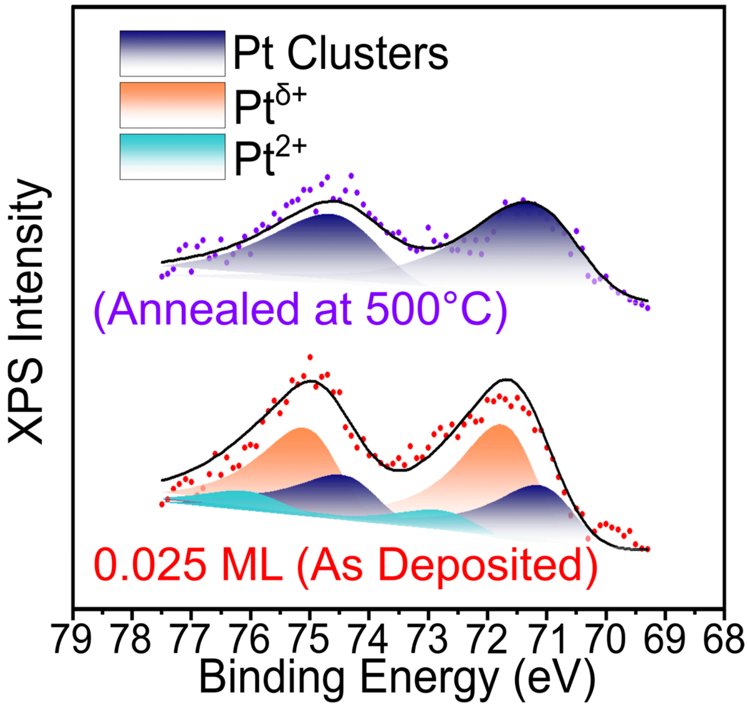

Figure S2. Pt 4f XPS spectra acquired before and after annealing of the 0.025 ML Pt/$Fe_2O_3(1\bar{1}02)$-(1×1) model catalyst to 500 °C. This results in significant thermal sintering and the formation of metallic-Pt particles on the surface. The resulting Pt $4f_{7/2}$ peak appears at 71.2 eV.

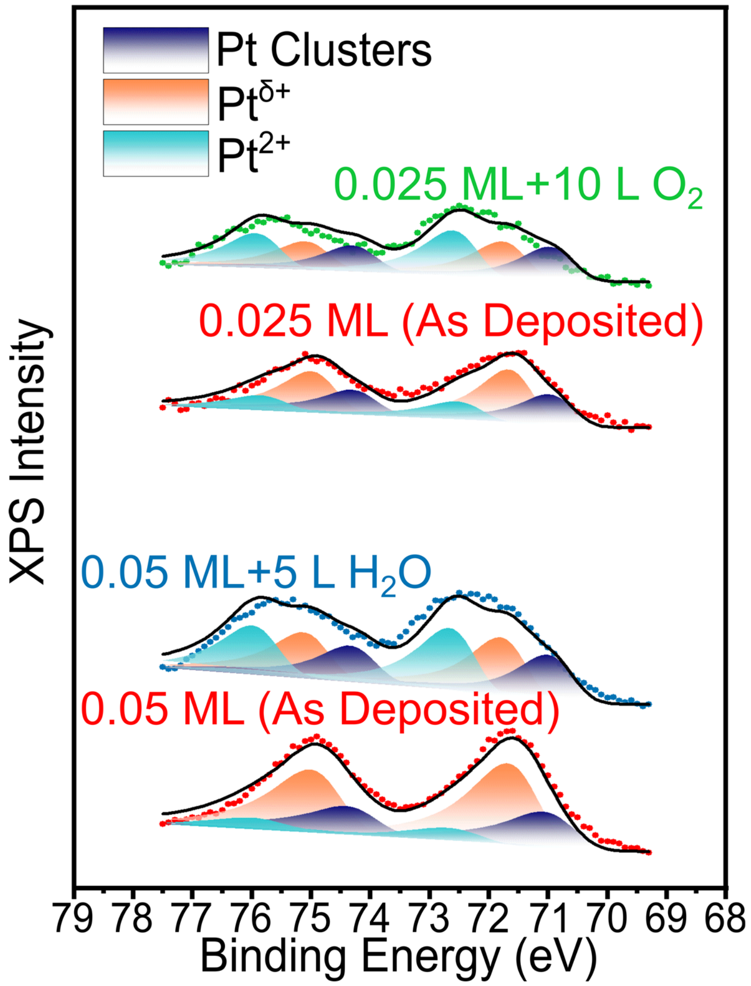

Figure S3. Pt 4f data acquired before and after exposing the 0.025 ML Pt/Fe_2O_3(1$\bar{1}$02)-(1×1) model catalyst to H_2O and O_2. Exposure to both molecules results in significant growth of the Pt^{2+} peak component at 72.7 eV at the expense of the Pt$^{\delta+}$ component at 71.8 eV.

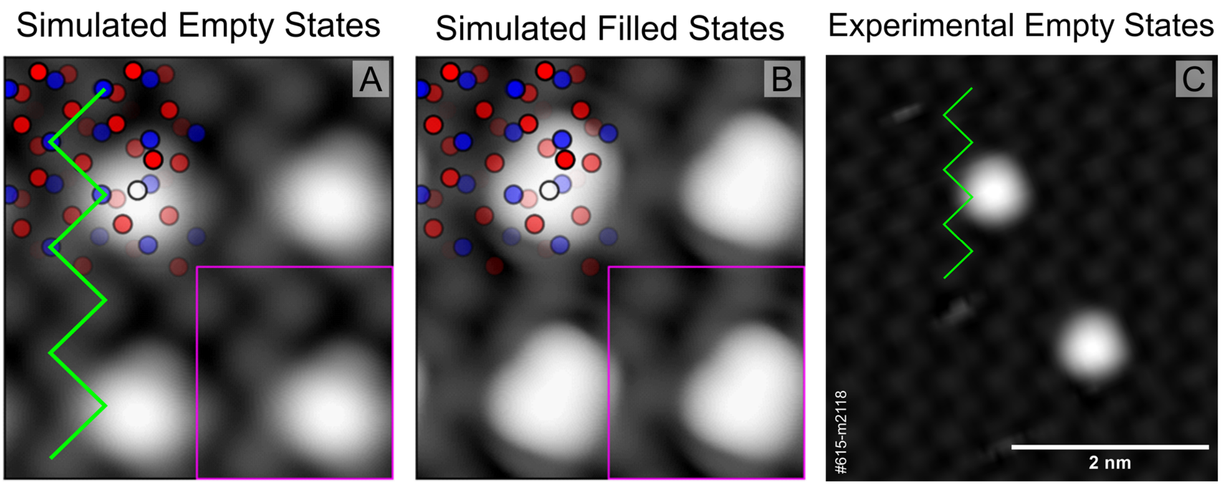

Figure S4. (A-B) Simulated STM images of the restructured Pt atom (surface O displaced) on α-$Fe_2O_3(1\bar{1}02)$-(1×1) as shown in Figures 4C and 4D of the main text. The structure is periodically repeated (the computational cell is indicated in pink) and the positions of atoms in the vicinity of the surface are indicated. The atom's distance from the surface is displayed via the transparency of the makers. We used the BSKAN code[2] to simulate constant-current STM images within the Tersoff–Hamann approximation[3]. Constant-current isosurfaces were extracted at bias voltages of +3 V and –3 V, to simulate tunneling from the tip into the surface (imaging empty states, see panel A) and from the surface to the tip (imaging filled states, see panel B), respectively. (C) Experimental STM image of 0.025 ML Pt vapour-deposited on $Fe_2O_3(1\bar{1}02)$-(1×1) at room temperature (U_{sample} = +3.0 V, I_{tunnel} = 0.35 nA).

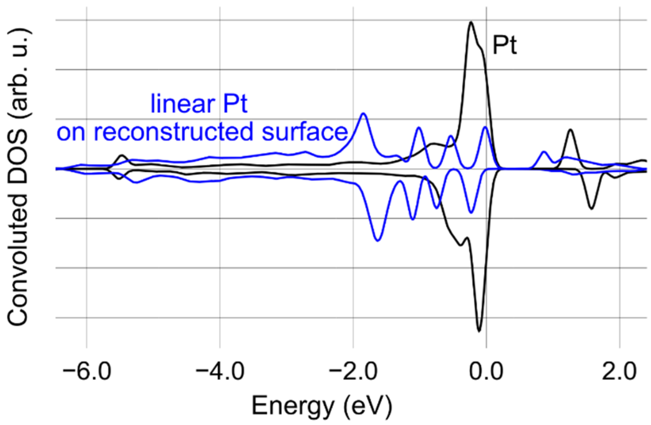

Figure S5. Electronic density of states (DOS) of the Pt adatom (black curve, corresponding to Figure 4A, B) and the pseudo-linear coordinated Pt (blue curve, corresponding to Figure 4C, D). The DOS shown is the sum of the projections on the Pt s, p, and d orbitals. The convolution with a Gaussian function (σ = 0.08 eV) provides easier visualization and can be rationalized as thermal broadening. The electronic DOS is lower in energy as the Pt adatom reaches its favored restructured site, stabilizing this structure. The Pt-adatom configuration serves as precursor state.

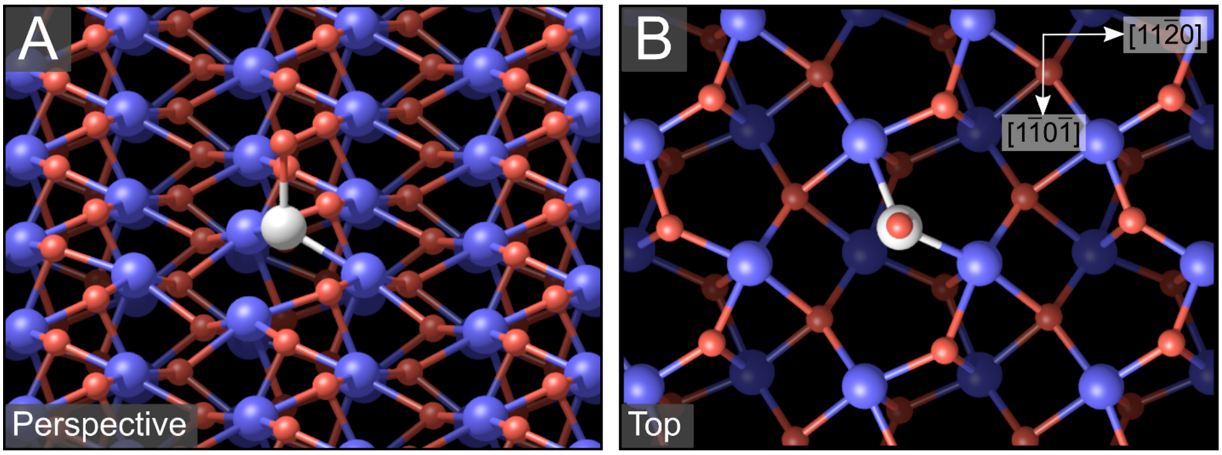

Figure S6. Structure model of the initial configuration for the CMA-ES search (the founder). To reduce clutter, the actual c(2×2) supercell used in CMA-ES searches is rendered here embedded in a larger bulk-terminated surface. Fe atoms are blue, O atoms are red, and the Pt is white.

Further Details Regarding The CMA-ES Computations

A major strength of the CMA-ES is its small number of hyperparameters. For all but two parameters, the founder (i.e., mean of the initial distribution) and the initial step size, we used recommended defaults that have been determined through extensive tuning on a set of benchmark problems available in the literature.[4] The rationale behind the choice of the founder was already described in the main text. For the initial step size, a value of 0.12 Å was used, in line with earlier works on structure search with CMA-ES.[5-6] Regarding convergence criteria, CMA-ES runs were terminated when the intra-generation standard deviation of the energy of individuals (candidate structures) remained below 0.05 eV for 15 successive generations.

The role of the CMA-ES is to suggest starting structures for local DFT relaxation, by steering structures toward different attraction basins of the potential-energy landscape without necessarily converging to any local minimum exactly. To select these starting structures from a CMA-ES evolution, we simply picked the overall lowest-energy structure from each run. In addition, some

further CMA-ES structures were hand-picked for relaxation based on visual inspection of the loss curve and identification of regions where a strong drop occurred over a few generations, which might indicate that a new, different basin of the potential-energy landscape had been visited (even if maybe just temporarily).

Movie S1. Time-lapse movie following the deposition of 0.025 ML of Pt on $Fe_2O_3(1\bar{1}02)$-(1×1). The actual duration of the movie is 120 minutes, with each scan frame taking about 3 minutes. The main text states that Pt single atoms are stable on the $Fe_2O_3(1\bar{1}02)$-(1×1) surface and rarely change their apparent height. To confirm this, a time-lapse movie consisting of 38 consecutive STM images of a ~50×50 nm^2 area on a large terrace of $Fe_2O_3(1\bar{1}02)$-(1×1) was created. The STM images were acquired under ultra-high vacuum (UHV) conditions. Over the course of 2 h, there were approximately 17 switches in the apparent height of atoms from high to low (highlighted with red circles) and 4 switches from low to high (highlighted with yellow circles). The time-lapse movie shows no significant aggregation of single atoms to form clusters.

References

1. Choi, J. I. J.; Mayr-Schmölzer, W.; Mittendorfer, F.; Redinger, J.; Diebold, U.; Schmid, M., The Growth of Ultra-Thin Zirconia Films on $Pd_3Zr(0001)$. *J. Phys. Condens. Matter* **2014**, *26*, 225003.
2. Hofer, W. A., Challenges and Errors: Interpreting High Resolution Images in Scanning Tunneling Microscopy. *Progr. Surf. Sci.* **2003**, *71*, 147-183.
3. Tersoff, J.; Hamann, D. R., Theory of the Scanning Tunneling Microscope. *Phys. Rev. B* **1985**, *31*, 805.
4. Hansen, N., The CMA Evolution Strategy: A Tutorial. *arXiv preprint arXiv:1604.00772* **2016**.
5. Arrigoni, M.; Madsen, G. K., Evolutionary Computing and Machine Learning for Discovering of Low-Energy Defect Configurations. *Npj Comput. Mater.* **2021**, *7*, 71.
6. Wanzenböck, R.; Arrigoni, M.; Bichelmaier, S.; Buchner, F.; Carrete, J.; Madsen, G. K., Neural-Network-Backed Evolutionary Search for $SrTiO_3(110)$ Surface Reconstructions. *Digital Discovery* **2022**, *1*, 703.
